# Biofilm Formation Is Crucial for Efficient Copper Bioleaching From Bornite Under Mesophilic Conditions: Unveiling the Lifestyle and Catalytic Role of Sulfur-Oxidizing Bacteria

**DOI:** 10.3389/fmicb.2021.761997

**Published:** 2021-10-22

**Authors:** Roberto A. Bobadilla-Fazzini, Ignacio Poblete-Castro

**Affiliations:** Biosystems Engineering Laboratory, Department of Chemical Engineering, Universidad de Santiago de Chile (USACH), Santiago, Chile

**Keywords:** *Leptospirillum* spp., *Acidithiobacillus thiooxidans*, biofilm, chalcopyrite, bornite

## Abstract

Biofilm formation within the process of bioleaching of copper sulfides is a relevant aspect of iron- and sulfur-oxidizing acidophilic microorganisms as it represents their lifestyle in the actual heap/dump mining industry. Here, we used biofilm flow cell chambers to establish laminar regimes and compare them with turbulent conditions to evaluate biofilm formation and mineralogic dynamics through QEMSCAN and SEM-EDS during bioleaching of primary copper sulfide minerals at 30°C. We found that laminar regimes triggered the buildup of biofilm using *Leptospirillum* spp. and *Acidithiobacillus thiooxidans* (inoculation ratio 3:1) at a cell concentration of 10^6^ cells/g mineral on bornite (Cu_5_FeS_4_) but not for chalcopyrite (CuFeS_2_). Conversely, biofilm did not occur on any of the tested minerals under turbulent conditions. Inoculating the bacterial community with ferric iron (Fe^3+^) under shaking conditions resulted in rapid copper recovery from bornite, leaching 40% of the Cu content after 10 days of cultivation. The addition of ferrous iron (Fe^2+^) instead promoted Cu recovery of 30% at day 48, clearly delaying the leaching process. More efficiently, the biofilm-forming laminar regime almost doubled the leached copper amount (54%) after 32 days. In-depth inspection of the microbiologic dynamics showed that bacteria developing biofilm on the surface of bornite corresponded mainly to *At. Thiooxidans*, while *Leptospirillum* spp. were detected in planktonic form, highlighting the role of biofilm buildup as a means for the bioleaching of primary sulfides. We finally propose a mechanism for bornite bioleaching during biofilm formation where sulfur regeneration to sulfuric acid by the sulfur-oxidizing microorganisms is crucial to prevent iron precipitation for efficient copper recovery.

## Introduction

One of the most important challenges that face the copper industry is the development of sustainable technologies to leach complex ores composed of mixed copper mineral species in low grades, with a prevalence of primary copper sulfides. The major component of these materials is usually chalcopyrite (CuFeS_2_), followed by bornite (Cu_5_FeS_4_) (Panda et al., 2015). To this end, bioleaching has emerged as an efficient technology to leach secondary sulfides under mesophilic conditions (ambient temperature) (Bustos et al., 1993) and primary sulfides mostly under thermophilic conditions above 60°C (Duarte et al., 1993) or at moderate thermophilic (45–50°C) in stirred tank bioreactors (Cancho et al., 2007; Hedrich et al., 2018). Recently, acidophilic bacterial consortia recovered 43% of copper from chalcopyrite using shake flasks at room temperature (25–30°C) (Ma et al., 2018). In another study utilizing a bioleaching column coupled with a fuel cell, the system yielded 244 mg L^–1^ of copper from 150 g of chalcopyrite at 30°C in 320 days (Huang et al., 2019).

In the case of bornite, studies have shown bioleaching activity under mesophilic conditions compared with abiotic controls (Jun et al., 2008; Bevilaqua et al., 2010). Besides, researchers have proposed a galvanic effect with chalcopyrite accelerating the oxidative dissolution of bornite in the presence of microorganisms (Wang et al., 2016). Inoculation of the moderate thermophilic *Leptospirillum ferriphilum and Acidithiobacillus caldus* in shaking flasks at 45°C enabled the conversion of bornite into several intermediates including covellite (CuS) and isocubanite (CuFe_2_S_3_) through the bioleaching process, with a final copper extraction of 70% (Hong et al., 2019).

Finally, several works have established the high bioleaching efficiency of thermophiles over chalcopyrite (Petersen and Dixon, 2002), but none of them uncovered the role of biofilm formation in this process. Recently, a study reported biofilm development over chalcopyrite under moderate thermophilic conditions, where *L. ferriphilum* was inoculated at a high concentration (above 10^8^ cells/g mineral) in the presence of under-stoichiometric iron concentrations (Bellenberg et al., 2018). A few studies have specifically addressed mesophilic biofilm formation on chalcopyrite, with researchers postulating that high precipitation of jarosite and elemental sulfur restrict biofilm development (Lei et al., 2009), while others identified microcolonies of *At. ferrooxidans* and *L. ferriphilum* forming a monolayered biofilm under short contacting periods (Africa et al., 2010).

Here, we demonstrated that mesophilic inoculation of bacteria with iron- and sulfur-oxidizing capacity in a flow cell chamber enables the detailed study of biofilm development on bornite resembling heap/dump bioleaching conditions when assuming a homogeneous packed bed behavior, where liquid percolation is driven by gravity through the ore particle surface. The biofilm-promoting laminar regime improved the copper leaching rate compared with stirred conditions in the presence of ferrous iron (Fe^2+^) and highlighted the importance of biofilm formation and its microbial composition to explain the mode of action and lifestyle of acidophiles carrying iron and sulfur oxidization during bornite bioleaching for copper extraction.

## Materials and Methods

### Consortium and Culture Conditions

The acidophilic consortium used in this study was obtained by enrichment from ore samples from mining regions in Chile. This consortium was maintained in batch aerated bubble column reactors at 30°C in modified 9K mineral salt medium [990 mg/L of (NH_4_)_2_SO_4_, 145 mg/L of NaH_2_PO_4_⋅H_2_O, 52 mg/L of KH_2_PO_4_, 100 mg/L of MgSO_4_⋅7H_2_O, and 21 mg/L of CaCl_2_] (Bobadilla-Fazzini et al., 2011), adjusted to pH 1.6 and containing soluble iron (5 g/L Fe^2+^), elemental sulfur (5 g S^0^/L), and chalcopyrite concentrate (0.2/L) as energy sources. The microbial composition analyses were performed by specific q-PCR determination as previously described (Bobadilla-Fazzini et al., 2011) indicated 76% abundance of *Leptospirillum* spp. and 24% of *Acidithiobacillus* spp.

### Mineral Samples

High-purity chalcopyrite (optical mineralogical characterization as 90.07% chalcopyrite, 0.10 covellite, 7.11% pyrite, and 2.72% unspecified gangue) and bornite (optical mineralogical characterization as 74.91% bornite, 15,89% Cc, 0.16% chalcopyrite, 0.79% digenite, 0.09% covellite, 0.35% rutile, 1.69% hematite, and 6.13% unspecified gangue) were obtained by manual picking from ore samples from the Geological Department of the Universidad de Chile. Samples were ground and sieved with 325 Tyler mesh, which means that all particles were below 0.044 mm.

### Screening Bioleaching Assays

Every mineral sample was attached to a plastic tape to cover a surface of approximately 1.5 × 1.5 cm registering the exact mass of mineral. The plastic tape with the bound mineral was attached to the bottom of a six-well plate with the mineral facing upward. Turbulent flow of culture medium (8 ml per well) was carried out using modified 9K medium either including ferric (0.7 g Fe^3+^/L) or ferrous iron (0.7 g Fe^2+^/L) as sulfate salts and incubating the plate at 30°C and 150 rpm under non-sterile conditions and including mesophilic inoculation. The acidophilic consortium was added to the solution to reach an initial density of 10^6^ cells/g mineral. For both conditions (with and without inoculation) three to five replicates were set up for every analyzed time. The solution obtained was directly sampled at the end of the assay without solid washing. Evaporation was prevented with a hermetic plastic cover on top of each six-well plate.

### Flow Cell Bioleaching Assays

A set of custom-made flow cells with a lower inlet and an overflow chamber was done in acrylic, recirculating a volume of 100–200 ml of culture minimal medium (Bobadilla-Fazzini, 2017). Every mineral sample was attached to a plastic tape in order to cover a surface of approximately 1.5 × 1.5 cm registering the exact mass of the mineral. The plastic tape with the bound mineral was attached to a glass slide that was placed on the top of the flow cell with the mineral facing downward. Flow was set at a free-stream fluid velocity of 0.02 cm/s and pipe diameter of 0.3 cm, where a laminar flow was assured (Reynolds number below 10) of culture-modified 9K medium with ferrous iron (0.7 g Fe^2+^/L) with a peristaltic recirculation pump and avoiding turbulent flow placing a bubble trap between the pump and the flow cell inlet. Mesophilic inoculation was done adding the acidophilic consortium to the solution to reach an initial density of 10^6^ cells/g of mineral just at the beginning of the incubation. All conditions were set up for every analyzed time in triplicate. All flow cells were incubated at 30°C under non-sterile conditions assuming solution saturation for oxygen and carbon dioxide. The recirculating solution was periodically sampled, and evaporation was compensated with pure water before each sampling time.

### Biofilm and Microbial Analysis

Measurements were done directly on mineral samples and solutions stopping three flow cell replicates for every condition and analyzed time. Cell concentration in the recirculating solution was done by direct chamber counting under phase-contrast microscope (Thoma Chamber, depth 0.010 mm). Further strain proportion was determined by specific q-PCR determination as previously described (Bobadilla-Fazzini et al., 2011). Briefly, purified genomic DNA was extracted and analyzed with total bacteria 16S rRNA gene region primers (forward 5′-GTGCCAGCMGCCGCGGTAA-3′, reverse 5′-CCGTCAATTCCTTTGAGTTT-3′), total Archaea 16S rRNA gene region primers (forward 5′-ACGGGGCG CAGCAGGCGCGA-3′, reverse 5′-YCCGGCGTTGAMTCCAA TT-3′), 16S rRNA gene for *Acidithiobacillus thiooxidans* (forward 5′-TAATATCGCCTGCTGTTGAC-3′, reverse 5′-TTTCACGACAGACCTAATG-3) and 16S rRNA gene for *Leptospirillum* spp. (forward 5′-TGAGGGGACTGCCAGCGAC-3′, reverse 5′-CTAGACGGGTACCTTGTTAC-3′). Alternatively, for biofilm analysis, previously designed fluorescent *in situ* hybridization (FISH) method and probes were used for *Leptospirillum* spp. (FISH-FAM: 5′-CGCTTCCCTC TCCCAGCCT-3′) (Bond and Banfield, 2001) and *Acidithiobacillus* spp. (5′-ACCAAACATCTAGTATTCATCG-3′) (Peccia et al., 2000).

### Chemical Analysis

Total iron and copper were determined by Atomic Absorption Spectrometry (Perkin Elmer Analyst 400), and Fe(II) by the o-phenantroline method (Kolthoff and Sandell, 1963).

### Mineralogical Analysis

For every sample, one section was dried overnight for mineralogical analysis, and the other section taken for SEM-EDS analysis (FEI Quanta 250) after sputter coating with gold. Mineralogical analysis was performed directly over the dry mineral samples at different times by bulk mineralogical analysis (BMA) with QEMSCAN Express equipment (FEI). The analytic methodology chosen was a combination of bulk mineral analysis with linear intercept method and a high-density field scan analysis (2.5 μm × 2.5-μm pixel size) with a minimum of 200,000 pixel analysis/sample in order to obtain a general mineral overview and a detailed mineral association mapping.

## Results and Discussion

**FIGURE 1:**
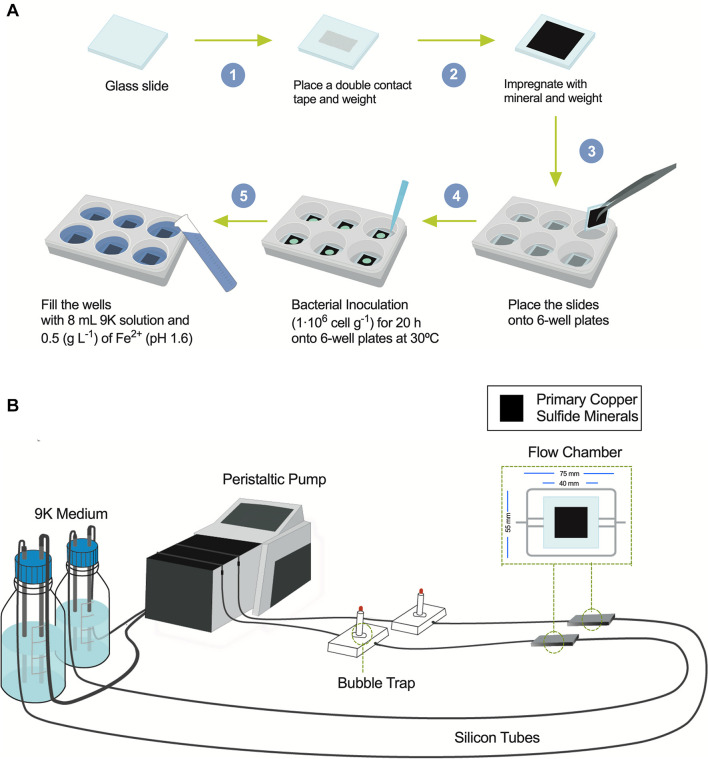
Diagrams showing the flow chamber for biofilm formation and the screening assay under shaking conditions. **(A)** Screening procedure scheme to bioleach copper from primary copper sulfides in mesophilic conditions (30°C) under shaking using six-well plates with inoculation of bacterial acidophiles. **(B)** Flow cell model and assembly diagram of bioleaching copper from primary copper sulfides under laminar regimes and buildup biofilm composed of acidophilic bacterial consortium growing at 30°C.

Mesophilic bioleaching has been used at an industrial scale for secondary copper sulfide ores, but not for the primary ones like chalcopyrite, given the low copper recovery and slow kinetics (Córdoba et al., 2008). For low-grade primary copper sulfide ores, a few industrial bioleaching applications are in the advanced stages to make the copper extraction economically feasible, to some extent, because of our inability to capture the lifestyle and growth development of the leaching microorganisms during copper recovery from actual primary ores. Thus, we investigated the capabilities of *Leptospirillum* spp. and *At. thiooxidans* to form biofilm on primary copper sulfides utilizing a standardized and reproducible flow cell approach that resembles heap/dump bioleaching conditions. It is important to highlight that most studies today employed primary copper sulfide high-purity minerals with concentrates added at low pulp densities and high cell loads in shaking flasks (Corkhill et al., 2008; Wang et al., 2016; Bellenberg et al., 2018). These growth conditions are optimal for homogeneous mixing and planktonic microbial growth, but certainly not for biofilm development (Tolker-Nielsen and Sternberg, 2011). First, we set the screening assay under turbulent flow regimes to mimic shaking flask liquid homogeneous conditions (Figure 1A). On the other hand, the method for studying bacterial biofilm development uses cell flow chambers under laminar regimes (Tolker-Nielsen and Sternberg, 2011; Crusz et al., 2012). Figure 1B depicts the components that comprise the cell chambers to study metal-leaching acidophiles. We found that the bubble trap is essential for attaining a homogeneous flow and proper propagation of acidophilic microorganisms (Crusz et al., 2012). We next placed on top of the cell chamber, in various sets of experiments, high-purity natural primary copper sulfide minerals to evaluate biofilm formation and copper extraction and finally compare it with turbulent conditions. Importantly, the minerals were not cleansed by acid solutions (HCl) nor autoclaved since these procedures alter the surface of the material, provoking initial leaching of copper and eliminating native microorganisms inhabiting these primary copper sulfide materials (Cerda et al., 2018).

### Chalcopyrite Bioleaching

**FIGURE 2:**
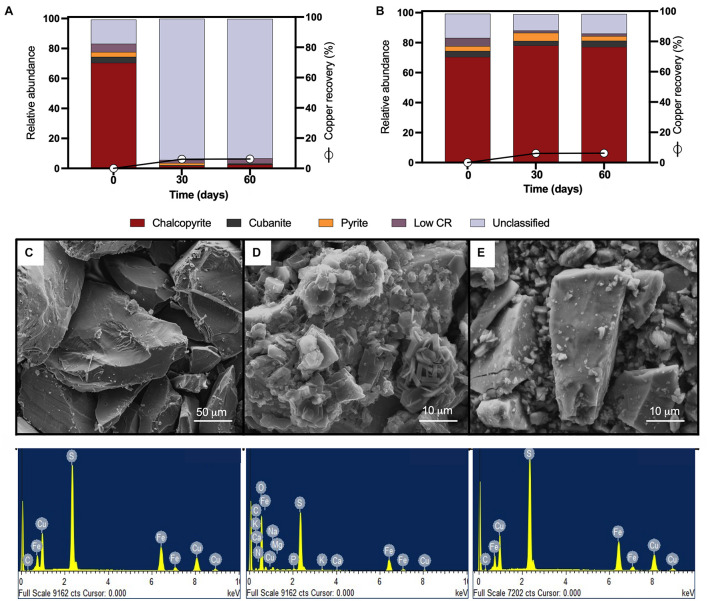
QEMSCAN-BMA analysis of high-purity natural chalcopyrite before bioleaching (t0) and after flow cell bioleaching assay (30 days) for **(A)** turbulent and **(B)** laminar conditions. **(C)** SEM image and EDS spectrum of high purity chalcopyrite before bioleaching. SEM images and EDS spectra of high-purity chalcopyrite after 30 days of **(D)** turbulent (screening method) and **(E)** laminar flow mesophilic bioleaching (flow cell method). Low CR: no mineral detection.

Mineralogical analyses were done on the head mineral to confirm chalcopyrite purity (Figure 2A, time 0) and at the end of the bioleaching assay to record possible transformations (Figure 2A, 30 and 60 days). Chalcopyrite inspection before bioleaching by QEMSCAN-BMA indicated a purity of 70% (previously estimated over 90% by optical microscopy), with a significant 16% of unclassified minerals (Figure 2A), with a clean surface as observed by SEM (Figure 2C). Under shaking conditions, precipitates completely covered the chalcopyrite surface after 30 days of bioleaching, partially classified as potassium and/or sodium jarosite based on the EDS elemental identification (Figure 2D). This was not the case for chalcopyrite bioleached under laminar regimes as the micrographs show low precipitates, and EDS analysis remained constant (Figure 2E).

Using the laminar flow chamber, the final mineralogical analysis showed significant variations with an increment in chalcopyrite proportion to 78% and reduced unclassified material to 11%, probably indicating the removal of initially present impurities from the mineral surface. On the contrary, in the turbulent flow regime, the mineralogical analysis was almost unable to identify chalcopyrite at the end of the assay (only 2% identification at day 30), while the unclassified raised to 94% attributed mainly to not identified mixed phases (Figure 2A, 30 and 60 days), indicating major mineral surface coverage by formed precipitates including potassium and/or sodium jarosite as indicated by EDS (Figure 2D). The screening bioleaching assay and the laminar flow condition showed a low copper recovery of 6% in 30 days incubated at 30°C and maintaining this copper level until day 60 (Figures 2A,B). Biofilm formation did not occur under turbulent or laminar flow regimes (Figures 2D,E). These results are in agreement with those previously published by Lei et al. (2009). Some studies describe biofilm buildup on the altered surface of the chalcopyrite in a short period where chalcopyrite was either previously electro-oxidized resulting in S^0^, CuS, and S^2–^ that promote bacterial attachment (García-Meza et al., 2013), or crushed down to a size of less than 80 μm and finally polished (Yang et al., 2015). Other works using batch stirred bioreactors set at 42°C and inoculated with *L. ferriphilum*, *Sulfobacillus* spp., and *A. caldus* showed 94% extraction of the available copper in the concentrate, where chalcopyrite remained intact during the leaching process (6% of the remaining Cu) (Spolaore et al., 2011; Hedrich et al., 2016). This confirms that even at moderate thermophilic conditions and employing a different acidophilic consortium from this study, it is challenging to leach the copper content from this primary sulfide mineral.

### Bornite Bioleaching

**FIGURE 3:**
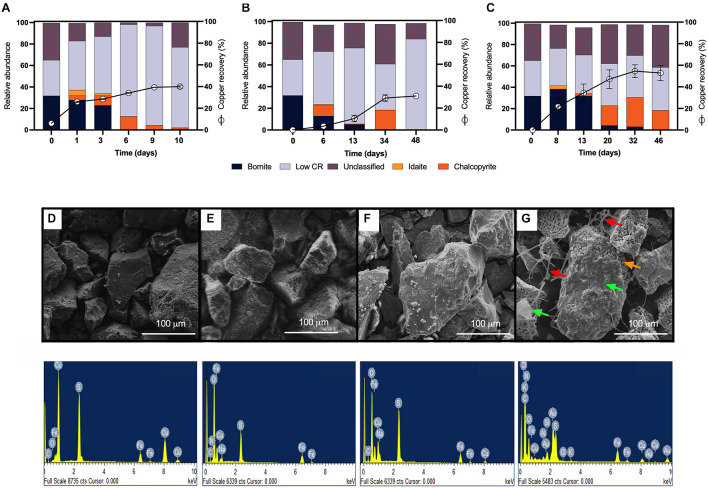
Mineralogical dynamics, SEM images, and EDS spectra of high-purity natural bornite bioleaching under mesophilic (30°C) inoculation. **(A)** Turbulent flow bioleaching with addition of ferric iron (screening method), **(B)** turbulent flow bioleaching with addition of ferrous iron (screening method), **(C)** laminar flow bioleaching (flow cell method). SEM images and EDS spectra for **(D)** bornite before bioleaching, **(E)** bornite after 10 days (40% Cu recovery) under turbulent flow mesophilic bioleaching (screening method), **(F)** bornite after 48 days (30% Cu recovery) under turbulent flow mesophilic bioleaching with addition of ferrous iron (screening method), **(G)** bornite after 46 days (54% Cu recovery) under laminar flow mesophilic bioleaching (flow cell method). Red, orange, and green arrows indicate exopolysaccharide (EPS) network, altered mineral, biofilm formation, respectively. Low CR: no mineral detection.

Considering that chalcopyrite is commonly associated with bornite in nature and that a galvanic couple between these two minerals has been described (Wang et al., 2016), the next step was to analyze the mesophilic bioleaching process correlated with highly pure bornite. We first inoculated the same proportion of the acidophilic bacterial consortium used for chalcopyrite along with the addition of ferric iron (0.7 g Fe^3+^/L), which enabled the rapid recovery of copper from bornite (40%) after 10 days of cultivation under stirring conditions (Figure 3A). Previous studies showed accelerated bornite leaching in the presence of external ferric iron addition (Hidalgo et al., 2019), and on the contrary, the screening bioleaching assays carried out with the addition of ferrous iron (0.7 g Fe^2+^/L) exhibited a copper extraction close to 30% after 48 days at 30°C (Figure 3B). In parallel, the laminar regime leached 54% of the Cu content at day 32, with no further Cu recovery until day 48 (Figure 3C), illustrating the importance of the process set up and bacterial attachment to accelerate copper bioleaching from bornite at 30°C.

**FIGURE 4:**
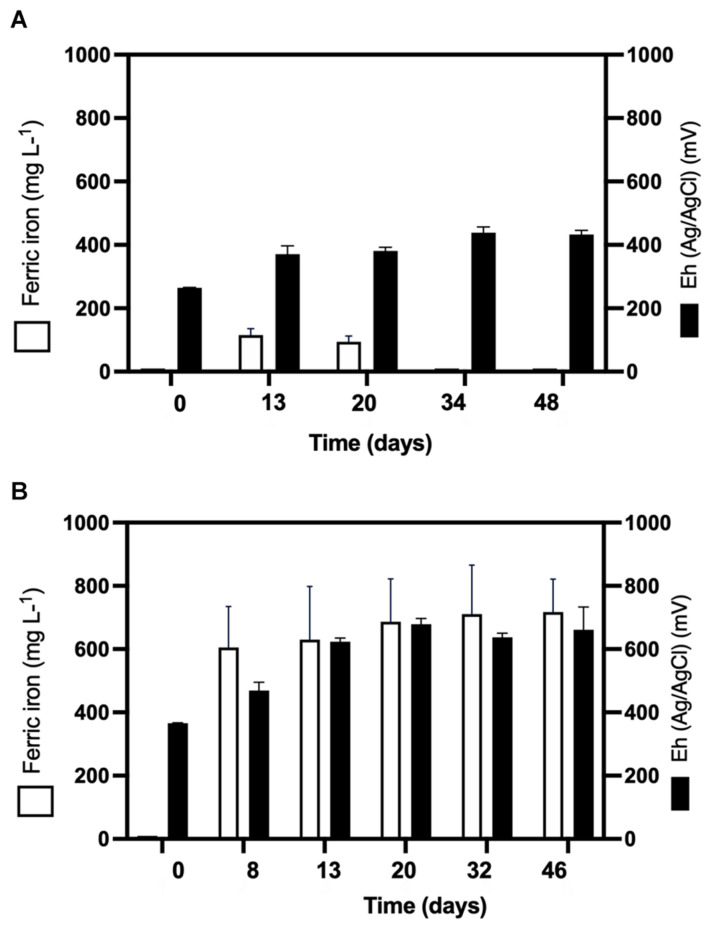
Ferric ion (Fe^3+^) formation and oxidation–reduction potential (Eh) during **(A)** turbulent flow bioleaching with addition of ferrous iron (700 mg/L) (screening method) and **(B)** laminar flow mesophilic bioleaching with addition of ferrous iron (700 mg/L) (flow cell method).

QEMSCAN-BMA recorded 32% bornite purity before bioleaching (estimated at 75% by optical microscopy) with an unclassified mineral fraction close to a third (time 0 in Figures 3A–C). The mineralogical dynamics of mesophilic bioleaching indicated in both regimens (turbulent and laminar flow) the intermediate formation of idaite (Cu_3_FeS_4_) in the first stage of bioleaching. Chalcopyrite detection was attributed to ferric ion precipitation masking further bornite/idaite detection (Figures 3A–C), though former studies consider it as part of the bornite (bio)leaching mechanism (Pal-ing, 2018). Turbulent and laminar flow regimes were initially inoculated including external ferrous iron (Fe^2+^) addition; in the turbulent regime, only a slight iron-oxidizing activity was observed until day 20 (Figure 4A) with a concomitant microbial growth, stopped by a significant pH increase (pH 4.25, data not shown) and consequent iron precipitation (Figure 4A). Interestingly, biofilm formation was observed only under laminar flow conditions, with a significant alteration in the bornite surface and an exopolysaccharide (EPS) network (Figure 3G, indicated with red arrows), compared with turbulent flow conditions where no biofilm formation was encountered with the initial addition of ferric nor ferrous iron (Figure 3F). Biofilm development mediated initially by EPS production during bioleaching depends on several factors including mineral type (Gehrke et al., 1998), process temperature (Zeng et al., 2010; Barahona et al., 2014), and the presence of organic compounds such as D-galactose (Saavedra et al., 2020) and sodium glucoronate (Bellenberg et al., 2015). For instance, pyrite is an excellent sulfide mineral for biofilm formation under acidic conditions as a strong electrochemical interaction occurs between the synthesized EPS-Fe^3+^ and the negatively charged mineral surface (Gehrke et al., 1998; Bellenberg et al., 2015). It differs highly compared with chalcopyrite, where EPS synthesis occurs only when inoculated at a high cell density (Lei et al., 2009; Yang et al., 2015; Bellenberg et al., 2018), with some studies using more than 10^9^ cells/g mineral as initial biomass (Lei et al., 2009). Unfortunately, this biomass loading is impractical at the industrial mining scale for cooper bioleaching of primary sulfur-bearing ores. It is clear that further investigation aiming at promoting biofilm development on chalcopyrite at mesophilic conditions is necessary under different flow regimes, varying the type and abundance of acidophilic consortia, resembling industrial operations, and taking care of the mineral characteristics, initial cell density, and avoiding major mineral pretreatments to make the process economically feasible.

### Microbial Analysis During Bornite Bioleaching

**FIGURE 5:**
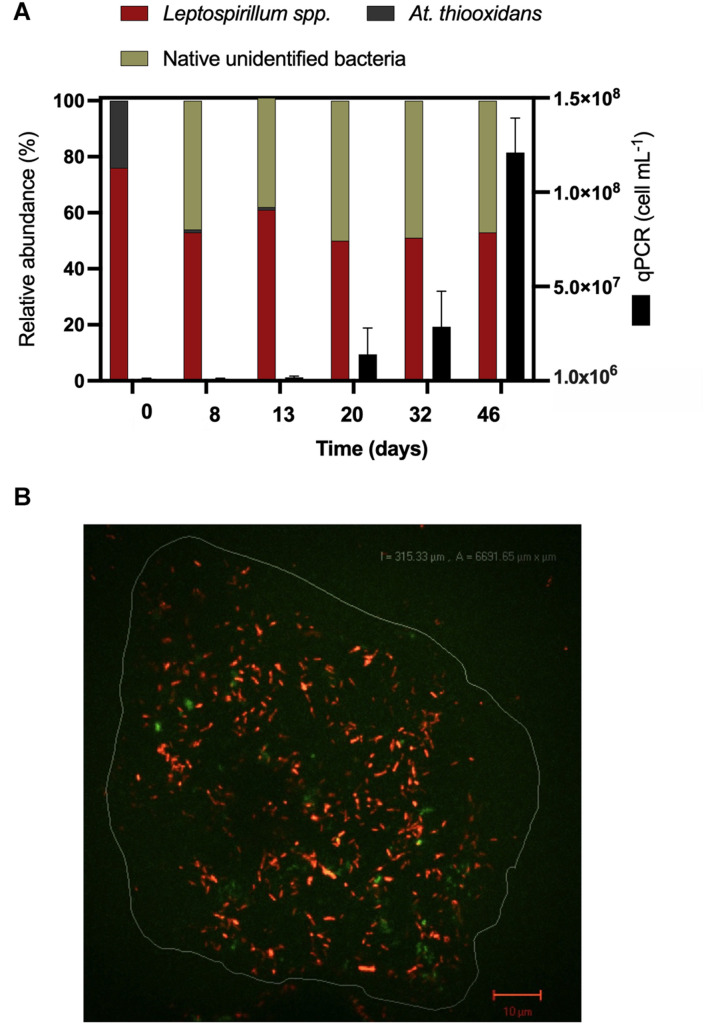
Microbiological determinations during high-purity natural bornite bioleaching under mesophilic (30°C) inoculation with external ferrous iron (Fe^2+^) addition in laminar flow bioleaching (flow cell method). **(A)** Microbial dynamics in the recirculating solution *via* qPCR and cell counts. **(B)** Representative biofilm composition by FISH (30 days) where the image corresponds to overlap of FISH-Cy3 (red, *Acidithiobacillus* spp.) and FISH-FAM (green, *Leptospirillum* spp.).

Considering the extensive copper recovery from bornite under the laminar flow regime, which was not seen here for the turbulent condition in the presence of ferrous iron (Fe^2+^), we next performed a dynamic microbial analysis during biofilm formation. Initial inoculation of the acidophilic consortium in a proportion 3:1 (*Leptospirillum* spp.:*At. thiooxidans*) was followed under the laminar flow condition, revealing the predominance of the iron oxidizers in solution until the end of the assay and the appearance of unidentified (possibly mineral native bacteria) in planktonic form (Figure 5A). After day 20, the iron present in the solution was detected in the ferric form (Figure 4B), correlating with the increment in the total iron-oxidizing bacteria in the recirculating solution (Figure 4B). However, FISH analysis indicated the prevalence of *Acidithiobacillus* spp. in the biofilm (Figure 5B), opening the discussion concerning the interaction and relevance of both planktonic and sessile microbial populations. On the one hand, the planktonic iron oxidizers recycle the ferric ion that actively (bio)leach the mineral surface, a phenomenon initially favored under turbulent conditions (Hong et al., 2019) based on the screening copper recovery kinetics observed (Figure 3). However, the lack of biofilm formation with the presence of microbes with sulfur-oxidizing activity did not generate the required acid production to balance its consumption during the microbial iron-oxidation process (Figure 4A), avoiding further copper extraction from bornite (Figure 3B). Clearly, this is not representative of the industrial heap/dump copper mining operations, where the kinetics of microbial iron and sulfur oxidation occur at a different pace. In this sense, the laminar flow biofilm analysis shows that the attached microbial population is mainly composed of sulfur-oxidizing bacterial species (Figure 5B), most likely due to mineral surface elemental sulfur accumulation (Lei et al., 2009; Bobadilla-Fazzini, 2017). This phenomenon can be considered useful in terms of initial mineral surface exposure to the ferric leaching agent, though detrimental due to extensive biofilm growth and mineral surface hindering at a later process stage, perhaps requiring specific conditions to tailor microbial composition for efficient copper extraction.

EDS analyses confirmed the expected bornite atomic composition before bioleaching (Figure 3D), and the altered bornite surface with the impoverishment of copper during bioleaching (Figure 3F), validating the identification of idaite by QEMSCAN. Moreover, EDS analysis showed that during the turbulent flow screening method, the precipitates formed over the surface of bornite appears to be sodium jarosite [Na Fe_3_(OH)_6_(SO_4_)_2_] due to notable increments in oxygen and sodium (Figure 3E), as recently reported for ferric sulfate pretreatment followed by moderate thermophilic bioleaching (Liu et al., 2020). On the other hand, the surface EDS analysis of laminar flow cell bornite showed no sodium at all but high carbon proportions indicating the organic nature of the biofilm formed (Figure 3G).

### Mechanism for Bornite Bioleaching During Biofilm Formation

**FIGURE 6:**
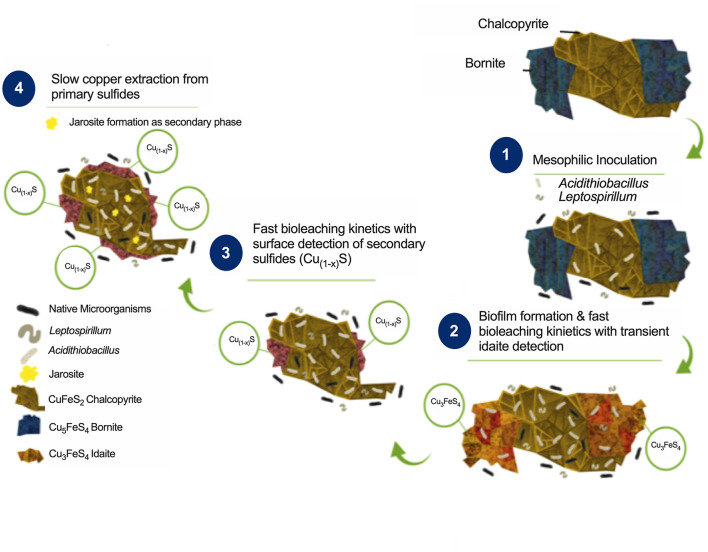
Proposed bioleaching mechanism for primary copper sulfides bornite and chalcopyrite under mesophilic inoculation of bacteria with iron- and sulfur-oxidizing activity forming biofilm.

Combining the biofilm development, ferric profile, and the mineralogical QEMSCAN and EDS analyses, it is possible to propose a mechanism for bornite bioleaching under laminar flow conditions (Figure 6). Previous studies have shown two stages for bornite ferric leaching (Pesic and Olson, 1984), with an initial leaching rate significantly dependent on ferric ion concentration and with no elemental sulfur formation leading to idaite:


(1) CuFeS5+44Fe⟶3+CuFeS3+42Cu+2+4Fe2+

Indeed, the ferrous iron oxidation is mediated by *Leptospirillum* spp. yielding the ferric form:


(2) 2Fe+2+1/2O+22H⟶+2Fe+3+HO2

This first stage is observed in our analysis from the beginning of the process until days 34 and 13 for turbulent and laminar flow with ferrous iron addition, respectively, with a corresponding copper extraction close to 30% (Figure 3). In the second stage, a slow leaching rate of idaite arises independent of ferric ion concentration (Figure 3C) where the sulfur produced is proportional to copper dissolved and tending to envelop the residue mineral particles, therefore, being the limiting step:


(3) CuFeS3+48Fe⟶3+S+03Cu+2+9Fe2+

Here, the iron- and sulfur-oxidizing microorganisms exert a catalyzing role, with the pH maintenance as a critical aspect in order to prevent ferric ion precipitation (Figure 4B), a role exerted by the sulfur-oxidizing biofilm controlling the leaching kinetics (Figure 5B):


(4) S+03O+22HO2⟶2SO4+2-4H+

These results confirm that mesophilic biofilm occurs extensively over bornite under laminar flow regimes, modifying its surface in a very different way than ferric leaching does under turbulent flow conditions (Hong et al., 2019; Liu et al., 2020). In addition, as the buildup of biofilm comprises several stages, initiating with the bacterial adherence to the surface, then the cells aggregate entering a phase of irreversible attachment, maturation, and finally dispersion (Garnett and Matthews, 2012). This process could explain the significantly higher copper recovery levels compared with shaking conditions (Figures 3B,C).

More precipitates are observed under turbulent conditions masking the proper quantification of the resulting iron sulfide compounds (Figure 3). Overall, the flow cell chamber is a more appropriated approach to studying biofilm formation and mineralogic dynamics that resemble the heap bioleaching of this abundant primary copper sulfide.

## Conclusion

The main copper minerals worldwide correspond to primary sulfides chalcopyrite and bornite, so it is crucial to understand the microbial variations affecting their efficient copper extraction. In this sense, this work shows that the standard approaches of bioleaching tests under stirring conditions prevent biofilm formation on bornite and chalcopyrite at 30°C. In addition, we determine that biofilm development is not feasible on chalcopyrite even under laminar flow mesophilic conditions, posing a great challenge for copper hydrometallurgy. Successfully, the biofilm-forming laminar regime improves almost twice the leached copper content of bornite compared with stirring conditions in the presence of ferrous iron (Fe^2+^), reducing the time from 48 to 32 days of cultivation. For the first time, we demonstrated that the main microorganisms comprising the biofilm development on bornite are sulfur-oxidizing bacteria, while the ferrous-oxidizing bacteria are in planktonic form. This lifestyle of acidophiles explains the accelerated pace of copper recovery during biofilm formation, where the action of sulfur-oxidizing bacteria maintains the low pH *via* sulfuric acid formation, thus, preventing iron precipitation. The next step toward sustainable copper hydrometallurgy is to develop a process that promotes biofilm formation on the surface of chalcopyrite under mesophilic or moderate thermophilic conditions to extract copper from this bulk material efficiently, pointing to the creation of novel acidophilic microbial consortia.

## Data Availability Statement

The raw data supporting the conclusions of this article will be made available by the authors, without undue reservation.

## Author Contributions

RB-F performed experiments, analyzed the data, and wrote the manuscript. IP-C analyzed the data and wrote the manuscript. Both authors contributed to the article and approved the submitted version.

## Conflict of Interest

The authors declare that the research was conducted in the absence of any commercial or financial relationships that could be construed as a potential conflict of interest.

## Publisher’s Note

All claims expressed in this article are solely those of the authors and do not necessarily represent those of their affiliated organizations, or those of the publisher, the editors and the reviewers. Any product that may be evaluated in this article, or claim that may be made by its manufacturer, is not guaranteed or endorsed by the publisher.
